# Study of Awareness, Enrollment, and Utilization of Rashtriya Swasthya Bima Yojana (National Health Insurance Scheme) in Maharashtra, India

**DOI:** 10.3389/fpubh.2015.00282

**Published:** 2016-01-07

**Authors:** Harshad Thakur

**Affiliations:** ^1^Centre for Public Health, School of Health Systems Studies, Tata Institute of Social Sciences, Mumbai, India

**Keywords:** health insurance, social health protection, awareness, enrollment, utilization, Rashtriya Swasthya Bima Yojana, Maharashtra, India

## Abstract

**Introduction:**

Government of India launched a social health protection program called Rashtriya Swasthya Bima Yojana (RSBY) in the year 2008 to provide financial protection from catastrophic health expenses to below poverty line households (HHs). The objectives of the current paper are to assess the current status of RSBY in Maharashtra at each step of awareness, enrollment, and utilization. In addition, urban and rural areas were compared, and social, political, economic, and cultural (SPEC) factors responsible for the better or poor proportions, especially for the awareness of the scheme, were identified.

**Methods:**

The study followed mixed methods approach. For quantitative data, a systematic multistage sampling design was adopted in both rural and urban areas covering 6000 HHs across 22 districts. For qualitative data, five districts were selected to conduct Stakeholder Analysis, Focused Group Discussions, and In-Depth Interviews with key informants to supplement the findings. The data were analyzed using innovative SPEC-by-steps tool developed by Health Inc.

**Results:**

It is seen that that the RSBY had a very limited success in Maharashtra. Out of 6000 HHs, only 29.7% were aware about the scheme and 21.6% were enrolled during the period of 2010–2012. Only 11.3% HHs reported that they were currently enrolled for RSBY. Although 1886 (33.1%) HHs reported at least one case of hospitalization in the last 1 year, only 16 (0.3%) HHs could actually utilize the benefits during hospitalization. It is seen that at each step, there is an increase in the exclusion of eligible HHs from the scheme. The participants felt that such schemes did not reach their intended beneficiaries due to various SPEC factors.

**Discussion and conclusion:**

The results of this study were quite similar to other studies done in the recent past. RSBY might still be continued in Maharashtra with modified focus along with good and improved strategy. Various other similar schemes in India can definitely learn few important lessons such as the need to improve awareness, issuing prompt enrollment cards with proper details, achieving universal enrollment, ongoing and prompt renewal, and ensuring proper utilization by proactively educating the vulnerable sections.

## Introduction

The present study was part of the multicountry research project by Health Inc. (“financing health care for inclusion”). It was a 3-year (May 2011–April 2014) collaborative research project. It explored how social exclusion restricts access to health services despite recent health financing reforms and how social health protection (SHP) can be increased. Research was conducted in Ghana, Senegal, and the Indian states of Maharashtra and Karnataka (1).

### Background

As a member state of World Health Organization (WHO), India is committed to achieve universal health coverage through the reforms of health financing systems (2). These reforms are essential as the use of private health care facilities forces below poverty line (BPL) households (HHs) toward more out-of-pocket expenditure, catastrophic payments, and/or neglect of the health (3, 4). Catastrophic health expenditure remains a cause of impoverishment in the majority of the HHs in India (5). As a part of improving access to health services and minimizing the economic impact, several state governments in India have launched their own state-specific health insurance schemes particularly for the economically vulnerable HHs. Schemes such as Aarogyasri in Andhra Pradesh and Yashaswini in Karnataka are key examples of state-specific health insurance schemes (6).

### RSBY in India and Maharashtra

As one of the many reforms, the Government of India launched Rashtriya Swasthya Bima Yojana (RSBY – National Health Insurance Scheme) in the year 2008 (7). The main objective of RSBY was to provide financial protection from catastrophic health expenses to the people working in the unorganized sector in India by covering more than 55 million BPL HHs and thus improve their access to health services.

In Maharashtra, the RSBY was initially launched in seven districts in late 2008 and was then gradually extended to the rest of the state (32 out of 35 districts). But recently in Maharashtra, the RSBY is suddenly being withdrawn in early 2014 as a new state-sponsored health insurance scheme, Rajiv Gandhi Jeevandayee Arogya Yojana (RGJAY), is being gradually started since 2010 and expanded to the entire state by 2013 year end (8, 9).

In majority of the states in India including Maharashtra, the Ministry of Labour is responsible for the implementation of RSBY. Both central and state governments contribute the entire premium in RSBY. Both private and public insurance companies participate in the insurance program. RSBY scheme depends on these insurance companies for involving the local governance structure and deploy strategies such as preenrollment campaign and information, education, and communication (IEC) activities through announcement and advertising at public places to inform targeted population. The tasks of IEC activities are often shifted to the third party administrators (TPAs). TPAs are the agencies acknowledged by Insurance Regulatory Authority of India. They carry out enrollment activities, print smart cards, process insurance claims, and do other administrative tasks. The enrolled people can seek cashless hospital care from the identified network of hospitals (both public and private).

### Studies on RSBY

The RSBY scheme was regarded for its excellent technical architecture and spirit to provide business opportunity to all stakeholders involved. The portability of usage of the scheme made it more efficient as compared with other state-specific health insurance schemes (10). Since inception in 2008, RSBY claims to have covered more than half of the targeted population. RSBY website (http://www.rsby.gov.in/Overview.aspx) as seen on April 28, 2014 shows that there are 68,472,226 HHs to be covered and 36,985,740 have been covered. However, recent studies on RSBY present inconsistent and contradictory findings. Various studies have shown that there is a substantial variation across the states and within the states (11–13). In Gujarat, it was seen that while the RSBY has managed to include the poor under its umbrella, it has provided only partial financial coverage. Nearly 60% of insured and admitted patients made out-of-pocket payments (14).

The enrollment rate in Maharashtra was found to be lower than the national average, and studies on RSBY raise concern over the success of the scheme (15). The secondary data analysis also indicates that the program implementation in the districts of Maharashtra is poor (13). Factors such as lack of accurate data for enrolling the BPL HHs and lack of infrastructure at the grass root level are reported as the key reasons. Since the enrollment is reported to be poor, many BPL HHs are excluded from RSBY.

### Rationale

The literature on RSBY by and large remains descriptive. There are very few scientific studies on RSBY in the state, and rigorous research evidence is lacking. On the one hand, Maharashtra state is replacing RSBY with RGJAY, and on the other hand, there are many other states in India continuing the RSBY scheme along with their own state-specific schemes. Thus, it becomes important to look at the performance and relevance of RSBY for Maharashtra.

In this context, there are important questions like what is the level of awareness, enrollment, and utilization pattern among BPL population regarding RSBY? Who are excluded from participation in RSBY in spite of being a BPL HH? What are the possible factors responsible for this? Considering the government plans to invest more in state-specific health insurance schemes and adaptation of this as key mechanism to finance health services, it becomes essential to explore the overall RSBY experience.

### Objectives

The objectives of the current paper are to assess the current status of RSBY in Maharashtra in terms of proportions covered at each step such as awareness, enrollment, renewal, having card, and utilization. In addition, attempt has been made to compare the proportions in the urban and rural areas and to identify the social, political, economic, and cultural (SPEC) factors responsible for the better or poor proportions at all the steps, especially for the awareness of the scheme.

## Materials and Methods

**Figure 1:**
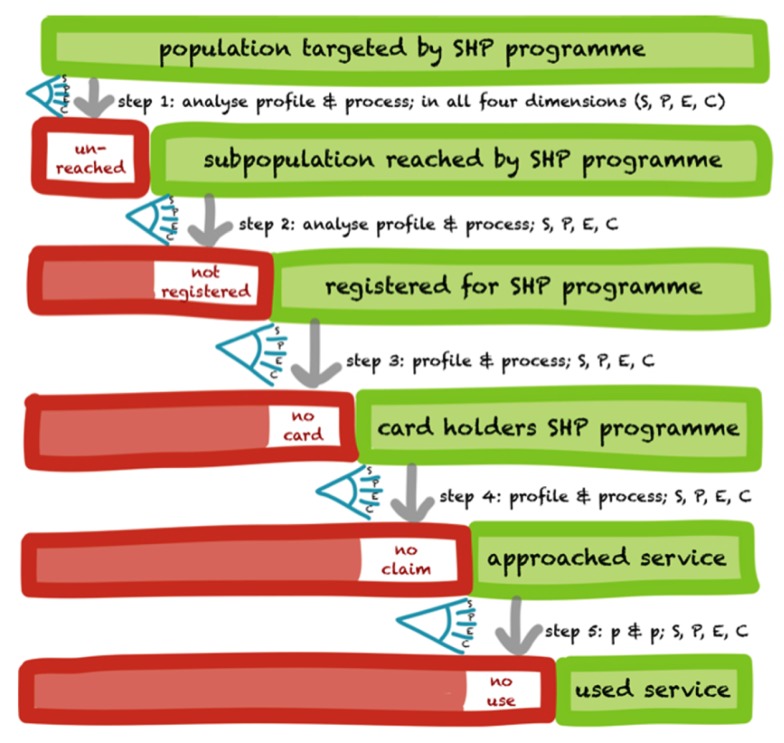
**The generic SPEC-by-step tool (developed by Health Inc.)**. Source: Ref. (16).

The research protocol was developed during December 2011 to April 2012. The Health Inc. developed a generic SPEC-by-steps tool/framework for assessing social exclusion as shown in Figure 1. This tool was used to identify the extent and nature of the exclusion at different levels and provides account of who is excluded/included (16). In constructing the tool, a generic SHP program is broken down into a series of steps, each step excluding a number of people. The excluded are shown on the left in a red box and the non-excluded on the right in a green box. At each step, SPEC factors are identified responsible for the exclusion/inclusion.

The study followed mixed methods approach. Initially, extensive review of literature was done followed by qualitative and quantitative methods to answer the research questions. The Institutional Ethics Committee’s permission was obtained, and written consents were taken from all the respondents.

### Settings

Maharashtra is located in the western and central part of India, with a coastline stretching nearly 720 km along the Arabian Sea. It is the second largest state in India both in terms of population and geographical area spread over 308,000 km^2^. The sample in Maharashtra was designed to provide estimates for the state as a whole, for urban and rural areas. Maharashtra is divided into 35 districts (administrative block below the level of state). The RSBY State Nodal Agency provided the information on the status of scheme and furnished the level of enrollment and duration of implementation, which further helped to narrow down on the study sites. The inclusion criterion for the districts in the study was minimum 2 years of scheme implementation. All 22 districts that met the inclusion criterion of at least 2 years of RSBY implementation were selected for this study.

**Figure 2:**
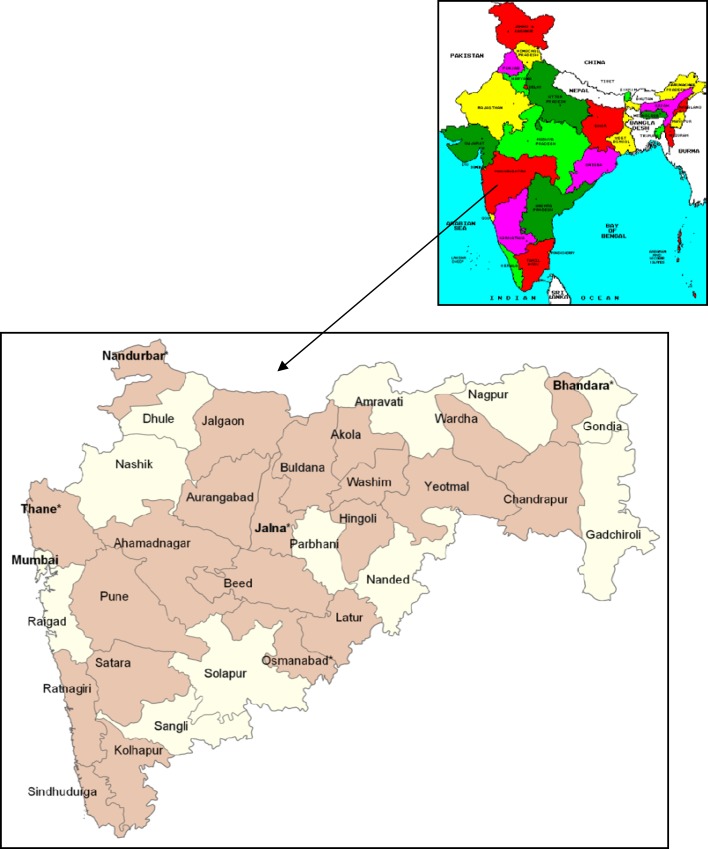
Map of India highlighting the location of Maharashtra and the selected districts in Maharashtra (Quantitative survey was carried out in 22 colored districts, and qualitative survey was carried out in five districts, shown using * sign and bold font.). Source: Wikimapia Commons and chooseindia.com.

The quantitative survey was carried out in all 22 districts. Five districts were selected for the qualitative survey. The selected districts are shown in Figure 2. The entire data collection was completed from December 2012 to February 2013.

### Quantitative Methods

This helped us to quantify the extent of the exclusion at each step of implementation. A target sample size of completed interviews with BPL HHs of 6000 was fixed. It was initially divided between urban and rural areas by allocating the sample proportionally to the population of these two areas. Within each of the sampling domains of rural and urban areas, a systematic, multistage stratified sampling design was used.

The Ministry of Labour had used the list of BPL HHs prepared in 2002 for the identification of beneficiaries during the implementation of RSBY. This list could have served as the sampling frame, but there were several problems with the BPL list. The list used by them was quite outdated with a gap of almost 10 years. Also, the list had many errors with poor quality of data entry, incompleteness, etc. (17, 18). So, it was decided to conduct the listing of BPL HHs on our own for the study. The HH listing process was carried out in the selected primary sampling units (PSUs) by the trained field investigators. The HH listing operation involved preparing up-to-date layout and sketch maps, assigning a number to each structure, identifying residential structures, and listing BPL HHs along with the names of heads of HHs.

The rural sample was selected in two stages. In the first stage, the selection of PSUs, which are villages or groups of villages (in the case of small linked villages), with probability proportional to population size (PPS) was carried out. A HH listing operation was carried out in each selected PSU to provide the necessary frame for selecting HHs at the second stage. The villages larger than 350 HHs were segmented into three or more segments (depending on the village size) of approximately equal size (usually about 100–200 HHs). From all the segments in each large village, two segments were selected randomly using the PPS method. HH listing was then done only in the two selected segments. Therefore, in all such large villages, the sampling design became a three-stage design. This was followed by the selection of 20 BPL HHs using systematic random sampling within each selected PSU in the next stage.

In the urban areas, a three-stage sampling procedure was followed as these areas are quite large. In the first stage, wards were selected with PPS. From each selected ward, one or two segments were selected with PPS in the second stage. Each segment comprised about 150–200 HHs. In the third stage, HH listing was carried out in the selected segment and 20 BPL HHs were selected using systematic random sampling.

### Quantitative Data Collection Process

For each HH, the head of the HH was interviewed through a structured interview schedule after their consent. If the HH was not available at that particular time, other members who were above the age of 16 years and were found knowledgeable were selected. The data collection was completed with the help of 40 trained field investigators in the selected regions. In order to address the issue of non-response at the HH level, a replacement strategy was adopted to achieve the target number of completed interviews. In case any selected HH could not participate in the survey for some reason, the next HH was selected for interview.

The questionnaire used was structured and was divided into 12 parts with different functions and objectives. It captured HH characteristics followed by awareness, enrollment, and utilization about RSBY. For our study, the HH was termed as “aware” if the respondent reported that he or she has seen the card. The “enrollment” was divided into two parts: ever (2010–2012) and current (2011–2012). The RSBY scheme provides a smart card that digitally identifies the users at the time of hospitalization and keeps a track of amount used from the total sum insured. Given its operational value, it was crucial for any poor HHs to possess the smart card. Whenever the beneficiary had used the smart card or taken the benefits of RSBY, it was considered as “utilization” of the scheme. The essential SPEC factors at each level were also captured.

Six thousand HHs (29,585 individuals) across 22 districts were covered through the quantitative HH survey in both rural and urban areas of Maharashtra.

### Qualitative Methods

For qualitative data, five districts were selected randomly from the five geographical regions allocated to different insurance companies by the state nodal agency implementing the scheme. The selected districts thus represented different geographical regions in Maharashtra as follows: Thane representing the western part of Maharashtra, Osmanabad representing the south, Jalna representing the central part, Bhandara representing the east, and Nandurbar representing the northern part. From each district, four PSUs were selected (two rural and two urban). In tribal districts (Nandurbar and Thane), one additional tribal PSU was selected for data collection. Qualitative data collection included stakeholder analysis, focused group discussions (FGD), and in-depth interviews (IDIs).

### Stakeholder Analysis

Stakeholder analysis was conducted in two parts. The first part consisted of a “stakeholder scoping” exercise that identified national and regional stakeholders for their motives, influence, and role in the RSBY scheme in Maharashtra. After understanding the stakeholders involved, IDIs of those who are closely related to the scheme were conducted. This included RSBY state nodal agency officer and the RSBY program officers of Insurance Company who were responsible for the implementation of RSBY scheme and the officials responsible for the RSBY enrollment camps. The findings were used to understand the issues in the program’s implementation.

### Focus Group Discussions

The purpose of FGD was to record the views of the socially excluded groups on the performance of RSBY and to identify the barriers they faced at each step on the basis of SPEC-by-step tool. From each selected PSU, one FGD was carried out. The HH list was used to identify the potential participants for discussion. At least 15–20 individuals from RSBY-enrolled and RSBY-non-enrolled HHs were identified ensuring representation of gender, different castes, religious groups, and the elderly. Overall, 18 FGDs were conducted.

### In-Depth Interviews with Household Members

In-Depth Interviews were conducted to describe the process of social exclusion based on the narratives of individual experience. The purpose was to understand the gravity of discrimination faced, social exclusionary practices, and perceived nature of the healthcare financing mechanism among those who are supposed to use them. Overall, this enabled us to understand why and how some individuals are excluded and how social exclusion affects health care utilization. The IDI respondents were selected from the FGD participants. The participants were a mix of individuals who had suffered some kind of illness within the past 1 year and were at different stages of the exclusion from the services. Interviews were conducted in the manner ensuring minimum disturbance. Overall, 34 IDIs were conducted with key informants.

### Data Analysis Using SPEC-by-Steps Tool and Statistical Methods

The hard copies of the questionnaire were screened for the completeness and accuracy of the responses. Once cleaned, the data were entered in the Statistical Package for Social Sciences (SPSS) version 15 for the quantitative data analysis. The HH data were initially analyzed using this localized SPEC-by-steps analytical framework. The analysis was carried at different steps with the stepwise denominator. This provided account of who is excluded or included at each step and helped to assess the efficacy of the RSBY implementation in Maharashtra. Later on, urban vs. rural and aware vs. not aware respondents were compared using common denominator. The two-way chi-square test and unpaired *t* test were applied while comparing different groups of respondents for different independent categorical variables. Whenever the *p* value was <0.05, it was considered as statistically significant at 95% confidence level.

ATLAS.ti was used for the analysis of qualitative data. The principle of grounded theory was used to code the transcripts. These findings helped to supplement the findings from the quantitative results and to identify the SPEC factors at each level. The findings were used to explain mechanisms associated with the low or high level of awareness, enrollment, and utilization about the scheme.

## Results

Initially, the results from the quantitative methods are presented, followed by the results from the qualitative methods.

### Results from the Quantitative Methods

#### Participants and Descriptive Data

While Table 1 presents the demographic and socioeconomic characteristics of selected urban and rural BPL HHs in Maharashtra, Table 2 presents the characteristics of individual population within the selected HHs. Majority of the participants are from the rural area (63.6% HHs and 62.3% individuals). The rural and urban HHs significantly differ from each other with regard to characteristics such as religion, caste, type of house, land holding, main economic activity of HH, and type of family. The urban and rural HH heads also significantly differ from each other with regard to sex and education.

**Table 1:** **Demographic and socioeconomic characteristics of selected households**.

Household characteristics	Rural (*N* **=** 3814)	%	Urban (*N* **=** 2186)	%	Total (*N* **=** 6000)	%	*p*-Value
Religion*							
Hindu	3211	84.2	1535	70.2	4746	79.1	<0.001
Muslims	224	5.9	330	15.1	554	9.2	
Buddhist and others	379	9.9	321	14.7	700	11.7	
Caste*							
Scheduled Caste (SC)	1115	29.2	890	40.7	2005	33.4	<0.001
Scheduled Tribes (ST)	738	19.3	420	19.2	1158	19.3	
Other Backward Castes (OBC)	1249	32.8	469	21.5	1718	28.6	
Others	712	18.7	407	18.6	1119	18.7	
**Household size**							
≤5	2533	66.4	1406	64.3	3939	65.7	0.100
>5	1281	33.6	780	35.7	2061	34.3	
Mean household size (SD)**	4.8 (2.3)		5.1 (2.3)		4.9 (2.3)		<0.001
Type of house*							
Kuchcha (temporary)	1374	36.0	690	31.6	2064	34.4	<0.001
Pucca (permanent)	551	14.5	521	23.8	1072	17.9	
Semi-pucca (semipermanent)	1889	49.5	975	44.6	2864	47.7	
Land holding*							
Yes	1526	40.0	132	6.0	1658	27.6	<0.001
No	2288	60.0	2054	94.0	4342	72.4	
Main economic activity of household*							
Self-employed (agriculture)	884	23.2	70	3.2	954	15.9	<0.001
Self-employed (non-agriculture)	206	5.4	272	12.4	478	8.0	
Agricultural laborers	1382	36.3	220	10.1	1602	26.7	
Casual laborers	966	25.3	1129	51.7	2095	34.9	
Regular wage/salary earning	287	7.5	457	20.9	744	12.4	
Others	89	2.3	38	1.7	127	2.1	
Type of family*							
Nuclear family	805	37.0	1342	41.3	2147	35.8	<0.001
Joint family	1254	57.1	2148	48.3	3402	56.7	
Single/extended	127	2.9	324	5.2	451	7.5	
Mean age of household head (SD)**	53 (13.2)		49 (13.0)		52 (13.3)		<0.001
Sex of household head*							
Male	3062	80.3	1682	76.9	4744	79.1	0.002
Female	752	19.7	504	23.1	1256	20.9	
Education of household head*							
Never went to school	1624	42.6	868	39.7	2492	41.5	<0.001
Primary (1–4)	843	22.1	421	19.3	1264	21.1	
Secondary (5–10)	1141	29.9	762	34.9	1903	31.7	
Higher secondary (11–12)	154	4.0	99	4.5	253	4.2	
Diploma, degree, and others	52	1.4	36	1.6	88	1.5	

*Two-way chi-square test is applied; *p* value is <0.05, so there is statistically significant difference.

**Unpaired *t* test is applied; *p* value is <0.05, so there is statistically significant difference.

**Table 2:** **Characteristics of individual population**.

Individual characteristics	Rural (*N* **=** 18,433)	%	Urban (*N* **=** 11,152)	%	Total (*N* **=** 29,585)	%	*p*-Value
**Sex**							
Male	9447	51.3	5595	50.2	15,042	50.8	0.072
Female	8986	48.7	5557	49.8	14,543	49.2	
Age group*							
0–14	4398	23.9	2928	26.3	7326	24.8	<0.001
15–59	11,425	62.0	7179	64.4	18,604	62.9	
60+	2610	14.2	1045	9.4	3655	12.4	
Mean age (SD)**	31 (20.7)		28 (19.0)		30 (20.1)		<0.001

*Two-way chi-square test is applied; *p* value is <0.05, so there is statistically significant difference.

**Unpaired *t* test is applied; *p* value is <0.05, so there is statistically significant difference.

#### Results from SPEC-by-Steps Tool

**Figure 3:**
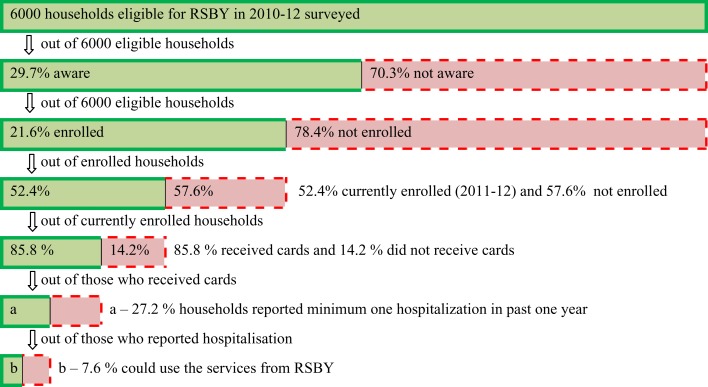
**Stepwise inclusion/exclusion of eligible population among the selected households of Maharashtra with stepwise denominator**.

The RSBY coverage is broken down into a cascade of steps as shown in Figure 3, with each step determining the number of people included and excluded by the program. There is a significant drop in the coverage of RSBY at each step of awareness, enrollment, and utilization. In Table 3, the denominator is fixed unlike Figure 3 and does not change at each step. Thus, if the denominator is kept constant, different picture is seen. It is seen that out of 6000 HHs, only 1781 (29.7%) HHs were aware; ever enrollment was seen in 1295 (21.6%) HHs and current enrollment was seen in 679 (11.3%) HHs for RSBY. At least one case of hospitalization in the last 1 year was reported in 1986 (33.1%) HHs, out of which 209 (3.5%) HHs were currently enrolled. Only 16 (0.3%) HHs could actually utilize the benefits during hospitalization. The rural coverage/performance is better (almost double or more) at each step as compared to the urban HHs and is highly significant.

**Table 3:** **Overview of RSBY performance in Maharashtra among selected households**.

Performance indicators at each step/level	Rural (*N* **=** 3814)	%	Urban (*N* **=** 2186)	%	Total (*N* **=** 6000)	%	*p*-Value
Aware households*	1316	34.5	465	21.3	1781	29.7	<0.001
Ever enrolled households*	1004	26.3	291	13.3	1295	21.6	<0.001
Currently enrolled households*	527	13.8	152	7.0	679	11.3	<0.001
Enrolled households with cards*	513	13.5	147	6.7	660	11.0	<0.001
Households with at least one hospitalization in last 1 year*	1210	31.7	776	35.5	1986	33.1	0.003
Among ever enrolled*	300	7.9	110	5.0	410	6.8	<0.001
Among currently enrolled*	149	3.9	60	2.7	209	3.5	0.018
RSBY utilization for at least one hospitalization*	14	0.4	2	0.1	16	0.3	0.046

*Two-way chi-square test is applied; *p* value is <0.05, so there is statistically significant difference.

The urban areas (35.5%) had more HHs with at least one hospitalization in the last 1 year compared to the rural areas (31.7%). But among the enrolled HHs, the rural areas (7.9%) had more HHs with at least one hospitalization in the last 1 year compared to the urban areas (5.0%). Both ever and currently enrolled HHs follow the same pattern. Utilization of RSBY benefits is also more among rural HHs as compared to urban. Table 4 presents comparison of HHs with total hospitalization episodes in the last 1 year among the selected HHs in the urban and rural areas. More HHs in the rural areas (41.1%) had hospitalization episodes compared to the urban areas (39.1%). Here, the difference between rural and urban HHs is statistically significant for ever enrolled HHs and RSBY utilization. If the denominator is taken as number of individuals, then the incidence of total hospitalization episodes in the last 1 year among total rural population (8.5%) is quite higher compared to urban population (7.7%).

**Table 4:** **Total hospitalization episodes in the last 1 year among the selected households**.

	Rural (*N* **=** 3814)	%	Urban (*N* **=** 2186)	%	Total (*N* **=** 6000)	%	*p*-Value
Total hospitalization episodes	1568	41.1	854	39.1	2422	40.4	0.120
Non-enrolled households	1179	30.9	699	32.0	1878	31.3	0.392
Ever enrolled households*	389	10.2	155	7.1	544	9.1	<0.001
Among currently enrolled households	215	5.6	100	4.6	315	5.3	0.076
RSBY utilization*	18	0.5	3	0.1	21	0.4	0.035

*Two-way chi-square test applied; *p* value is <0.05, so there is statistically significant difference.

The use of public or private hospital for hospitalization episodes in the last 1 year among the selected HHs was also studied. It was seen that more private hospitals (66.5%) were used as compared to public hospitals. In the rural areas, the use of private hospitals was quite high (70.7%) as compared to the urban areas (58.9%). The same pattern was seen among both enrolled and non-enrolled HHs.

#### Awareness About RSBY

The determinants of poor awareness about RSBY were studied further. The aware and not aware HHs were compared. Table 5 shows that the awareness is less in the urban areas (26.1%) compared to the rural areas (40.8%), and it is statistically significant. Table 6 presents the comparison between aware and not aware HHs among the urban and rural areas for demographic and background characteristics. There is significant statistical difference for religion, caste, HH size, economic activity, type of family, and sex and education of the HH head. For the type of house and landholding, the statistically significant difference is not present when compared between overall aware vs. not aware groups but present when compared between urban and rural areas in aware and not aware groups. Table 7 presents a comparison of SPEC dimensions/factors among the aware and not aware HHs among the urban and rural areas. For analyzing the factors associated with awareness, key variables based on the social exclusion framework were used. It is seen that the political factors are more significantly related to awareness as compared to social/cultural and economic factors in both the urban and rural areas.

**Table 5:** **Comparison of place of residence among the aware and not aware households**.

Place of residence*	Aware (*N* **=** 1781)	%	Not aware (*N* **=** 4219)	%	Total (*N* **=** 6000)	%	*p*-Value
Rural	1316	73.9	2498	59.2	3814	63.6	<0.001
Urban	465	26.1	1721	40.8	2186	36.4	

*Two-way chi-square test is applied; *p* value is <0.05, so there is statistically significant difference.

**Table 6:** **Comparison of demographic and background factors among the aware and not aware households as per their urban and rural background**.

Demographic and background characteristics	Aware				Not aware			

	Urban (*N* **=** 465), *N* (%)	Rural (*N* **=** 1316), *N* (%)	Total (*N* **=** 1781), *N* (%)	*p*-Value	Urban (*N* **=** 1721), *N* (%)	Rural (*N* **=** 2498), *N* (%)	Total (*N* **=** 4219), *N* (%)	*p*-Value
Religion*^,#^								
Hindu	267 (57.4)	1048 (79.6)	1315 (73.8)	<0.001	1268 (73.7)	2163 (86.6)	3431 (81.3)	<0.001
Muslim	113 (24.3)	103 (07.8)	216 (12.1)		217 (12.7)	121 (04.8)	338 (08.0)	
Buddhist and others	85 (18.3)	165 (12.5)	250 (14.0)		236 (13.7)	214 (08.6)	450 (10.7)	
Caste categories*^,#^								
SC	179 (38.5)	428 (32.5)	607 (34.1)	<0.001	711 (41.3)	687 (27.5)	1398 (33.1)	<0.001
ST	48 (10.3)	246 (18.7)	294 (16.5)		372 (21.6)	492 (19.7)	864 (20.5)	
OBC	133 (28.6)	422 (32.1)	555 (31.2)		336 (19.5)	827 (33.1)	1163 (27.6)	
Others	105 (22.6)	220 (16.7)	325 (18.2)		302 (17.5)	492 (19.7)	794 (18.8)	
**Household size**								
≤5	282 (60.6)	855 (65.0)	1137 (63.8)	0.095	1124 (65.3)	1678 (67.2)	2802 (66.4)	0.208
>5	183 (39.4)	461 (35.0)	644 (36.2)		597 (34.7)	820 (32.8)	1417 (33.6)	
Mean household size (SD)**^,#^	5.44 (2.739)	4.95 (2.204)	5.08 (2.364)	<0.001	5.01 (2.148)	4.77 (2.321)	4.87 (2.255)	<0.001
Type of house*								
Temporary	130 (28.0)	459 (34.9)	589 (33.1)	<0.001	560 (32.5)	915 (36.6)	1475 (35.0)	<0.001
Permanent	129 (27.7)	188 (14.3)	317 (17.8)		392 (22.8)	363 (14.5)	755 (17.9)	
Semipermanent	206 (44.3)	669 (50.8)	875 (49.1)		769 (44.7)	1220 (48.8)	1989 (47.1)	
Land holding*								
Yes	25 (05.4)	464 (35.3)	489 (27.5)	<0.001	107 (06.2)	1062 (42.5)	1169 (27.7)	<0.001
No	440 (94.6)	852 (64.7)	1292 (72.5)		1614 (93.8)	1436 (57.5)	3050 (72.3)	
Main economic activity of household*^,#^								
Self-employed (agriculture)	22 (04.7)	268 (20.4)	290 (16.3)	<0.001	48 (02.8)	616 (24.7)	664 (15.7)	<0.001
Self-employed (other)	87 (18.7)	70 (05.3)	157 (08.8)		185 (10.7)	136 (05.4)	321 (07.6)	
Agriculture labor	52 (11.2)	527 (40.0)	579 (32.5)		168 (09.8)	855 (34.2)	1023 (24.2)	
Casual labor	213 (45.8)	327 (24.8)	540 (30.3)		916 (53.2)	639 (25.6)	1555 (36.9)	
Regular wage/salary	84 (18.1)	95 (07.2)	179 (10.1)		373 (21.7)	192 (07.7)	565 (13.4)	
Others	7 (01.5)	29 (02.2)	36 (02.0)		31 (01.8)	60 (02.4)	91 (02.2)	
Type of family*^,#^								
Nuclear family	158 (34.0)	466 (35.4)	624 (35.0)	0.034	647 (37.6)	876 (35.1)	1523 (36.1)	0.001
Joint family	292 (62.8)	769 (58.4)	1061 (59.6)		962 (55.9)	1379 (55.2)	2341 (55.5)	
Single/extended	15 (03.2)	81 (06.2)	96 (05.4)		112 (06.5)	243 (09.7)	355 (08.4)	
Mean age of household head (SD)**	50.69 (13.040)	52.48 (13.058)	52.01 (13.073)	0.011	48.81 (12.906)	53.89 (13.429)	51.82 (13.344)	<0.001
Sex of household head*^,#^								
Male	377 (81.1)	1077 (81.8)	1454 (81.6)	0.715	1305 (75.8)	1985 (79.5)	3290 (78.0)	0.005
Female	88 (18.9)	239 (18.2)	327 (18.4)		416 (24.2)	513 (20.5)	929 (22.0)	
Education of household head*^,#^								
Never went to school	150 (32.3)	524 (39.8)	674 (37.8)	0.019	718 (41.7)	1102 (44.1)	1820 (43.1)	0.045
Primary (1–5)	140 (30.1)	397 (30.2)	537 (30.2)		415 (24.1)	653 (26.1)	1068 (25.3)	
Secondary (6–10)	140 (30.1)	316 (24.0)	456 (25.6)		488 (28.4)	617 (24.7)	1105 (26.2)	
Higher secondary (11th and 12th)	24 (05.2)	50 (03.8)	74 (04.2)		73 (04.2)	87 (03.5)	160 (03.8)	
Diploma and graduate	11 (02.4)	29 (02.2)	40 (02.2)		27 (01.6)	39 (01.6)	66 (01.6)	

*Two-way chi-square test is applied; *p* value is <0.05, so there is statistically significant difference for either/both urban and rural areas.

**Unpaired *t* test is applied; *p* value is <0.05, so there is statistically significant difference for either/both urban and rural areas.

*^#^Statistically significant (*p* < 0.05) when compared between aware vs. not aware groups*.

**Table 7:** **Comparison of social/cultural, political, and economic dimensions/factors among the aware and not aware households as per their urban and rural background**.

Background characteristics	Aware				Not aware			
	
	Urban (*N* **=** 465), *N* (%)	Rural (*N* **=** 1316), *N* (%)	Total (*N* **=** 1781), *N* (%)	*p*-Value	Urban (*N* **=** 1721), *N* (%)	Rural (*N* **=** 2498), *N* (%)	Total (*N* **=** 4219), *N* (%)	*p*-Value
**Social/cultural dimensions/factors**								
1. Participation in social organization	93 (20.0)	283 (21.5)	376 (21.1)	0.494	332 (19.3)	468 (18.7)	800 (19.0)	0.651
2. Discusses intimate and personal matters in the community^#^	415 (89.2)	1189 (90.3)	1604 (90.1)	0.495	1520 (88.3)	2203 (88.2)	3723 (88.2)	0.897
3. Feel that have been treated fairly because of your political beliefs, religion	453 (97.4)	1277 (97.0)	1730 (97.1)	0.670	1673 (97.2)	2424 (97.0)	4097 (97.1)	0.741
4. How often do you attend religious gatherings?*								
Once a month or more	28 (06.0)	98 (07.4)	126 (07.1)	0.042	93 (05.4)	157 (06.3)	250 (05.9)	<0.001
On holidays	282 (60.6)	813 (61.8)	1095 (61.5)		1048 (60.9)	1557 (62.3)	2605 (61.7)	
Marriages/funerals	85 (18.3)	268 (20.4)	353 (19.8)		340 (19.8)	556 (22.3)	896 (21.2)	
Never	70 (15.1)	137 (10.4)	207 (11.6)		240 (13.9)	228 (09.1)	468 (11.1)	
**Political dimensions/factors**								
1. Voted in recent election*^#^	456 (98.1)	1301 (98.9)	1757 (98.7)	0.201	1667 (96.9)	2457 (98.4)	4124 (97.7)	0.001
2. Participation in local politics*^#^	221 (47.5)	773 (58.7)	994 (55.8)	<0.001	516 (30.0)	1171 (46.9)	1687 (40.0)	<0.001
3. Political contacts*^#^	209 (44.9)	733 (55.7)	942 (52.9)	<0.001	625 (36.3)	1194 (47.8)	1819 (43.1)	<0.001
4. Contested in local election*^#^	6 (01.3)	46 (03.5)	52 (02.9)	0.015	8 (00.5)	53 (02.1)	61 (01.4)	<0.001
5. Member of political party^#^	17 (03.7)	51 (03.9)	68 (03.8)	0.832	32 (01.9)	70 (02.8)	102 (02.4)	0.050
**Economic dimensions/factors**								
1. Someone in the family a bank account holder	315 (67.7)	906 (68.8)	1221 (68.6)	0.660	1143 (66.4)	1715 (68.7)	2858 (67.7)	0.126
2. Had enough food in past 1 month^#^	401 (86.2)	1139 (86.6)	1540 (86.5)	0.865	1533 (89.1)	2246 (89.9)	3779 (89.6)	0.383
3. Aware of any scheme for BPL*	350 (75.3)	1090 (82.8)	1440 (80.9)	<0.001	1321 (76.8)	2027 (81.1)	3348 (79.4)	0.001

*Two-way chi-square test is applied; *p* value is <0.05, so there is statistically significant difference for either/both urban and rural areas.

*^#^Statistically significant (*p* < 0.05) when compared between aware vs. not aware groups*.

Though many respondents identified the scheme when showed a sample RSBY card, very few knew the scheme by its name. In fact, some respondents identified the RSBY card as either “Hospital Card” or “Insurance Card” or even “Aadhaar Card” (a unique identity card provided by the Government of India). At some places, the participants did not know about the scheme at all. In some other places, the awareness was better; however, detailed information was known to very few individuals. The depth of awareness was examined among the 1295 enrolled HHs. Among them, only 289 (22.3%) felt that they had received adequate information about RSBY. The majority had incomplete information on scheme’s benefits (such as services covered, sum insured, and empaneled hospitals), which are major barriers in the utilization of the benefits.

### Results from the Qualitative Methods

#### FGD and IDI Responses

The qualitative data (especially FGD and IDI) were helpful to ascertain the mechanism and reasons for the poor awareness, enrollment, and utilization of RSBY. It is also worth including some remarks made by the participants in their own words.

Some HHs were unaware and never enrolled in the scheme even though the enrollment was quite high in the neighborhood. Regarding this, one of the IDI respondents mentioned that *“Yes, they (neighbors) do not share valuable information. They feel that if they are getting benefits of welfare programmes, then why to bother about the rest of the households.” (Source: Jalana urban IDI)*.

Narrating the information exchange during the enrollment, one of the FGD participant said – *“(*… *we were told that* …*) this booklet consists of the names of hospitals (and services) which can be used for the hospitalization (services); we were not told in person about the card except that (*… *all the functions of the card and* …*) facilities are explained in the booklet (and one can read and understand them from it).” (Source: Nandurbar rural tribal FGD)*. Thus, only booklets were distributed during the enrollment. Many of the illiterate or less educated individuals might not be able to understand it.

Some respondents felt that they were deliberately excluded from many schemes including RSBY because they are not politically well connected: *“They (politically well connected families) received wells, land. They (government agencies) provide employment to those who already have enough and let the poor die.” “Yes! That is it! They (RSBY enrollment agencies) will come there (in the Gram Panchayat) and leave from there. They will not come here.” (Rural IDI woman whose husband was hospitalized and who was not enrolled)*.

From FGDs and IDIs, it emerged that the enrollment was usually done on a particular day by the enrollment agencies. There was no strategy to inform potential beneficiaries about this. For many respondents, it was not affordable to miss the work and daily wages (especially, the casual laborers). This was the reason why many HHs were being excluded from enrollment. *“The problem was they (people of enrollment agency) had not informed us before coming. So, when they came, we were not there. Then they came again, on another day. But they had not informed us. So, we couldn’t make it again* …*” (Source: Male participant from rural FGD in Nandurbar). “They (people of enrollment agency) informed people in the village (through the village head – sarpanch) that there will be a card distribution and everyone whose name features in the list are requested to stay back for the getting the card (enrollment). Some households did not listen to it (or were not able to understand it) and went on to work (to various places out of the village) thus they were not able to get the cards.” (Source: Bhandara rural IDI*). The awareness and enrollment were usually done simultaneously resulting in poor awareness among the respondents. Many HHs also failed to renew their membership. Further, those who renewed their membership were not necessarily provided with a new card. In some instances, the cards were left in the gram panchayat and not distributed or were renewed by overwriting on the older cards.

Even if the HH was enrolled for the last few years, there was uncertainty about which hospitals can provide services. *“Now how we will be aware? We never went there (to hospitals, clinics* …*) with the intention to use the card. In addition to that I have been to several hospitals for treatment and there is no relief (cure). Now you only can tell me where should I go for treatment? If there is proper information, only then we will go the hospital.” (Source: Household head – woman and a casual laborer from rural district of Bhandara)*.

Due to lack of awareness, several respondents had incurred expenditure for hospitalization episodes in spite of being covered by the scheme. *“(I didn’t have the right information about the scheme. Otherwise I would have saved the money that I spent on these two hospitalization episodes).” (Source: Jalgaon urban FGD respondent)*. Few individuals who utilized services of RSBY hospitals reported that there was demand for additional payments, pressure to purchase medicines from out of pocket, etc.

The results from the qualitative data also revealed that the poor awareness about the way RSBY functions must have led to poor enrollment and utilization by the HHs. The level of awareness was poor even among the HHs enrolled in the scheme. There was insufficient information exchange between the enrollment agencies and target HHs. Lack of effective IEC activities from the agencies, which were entrusted to enroll the HHs in the given district, emerged as a principal reason for the low awareness, enrollment, and utilization of the scheme.

The participants also felt that such schemes did not reach their intended beneficiaries due to various factors (e.g., illiteracy, poverty, poor planning, improper implementation, neglect of vulnerable people, etc.) and existence of a nexus of greed, corruption, and political power. Other important reasons that emerged from qualitative data are physical (differential geographical coverage for rural/urban/tribal areas), social/cultural (excluded groups such as SC/ST/OBC, women, elderly, children, minorities like Muslim in some areas), economical (denial of benefits to BPL families, leakage of benefits to well-off families), and political (policy makers and implementers not interested in implementation).

#### Stakeholder Responses

The key informants and stakeholders also reinforced these findings. The following key findings emerged from their responses:

• There are many such schemes simultaneously operating across India, which creates confusion for people as well as implementers.
• The strategies and efforts for creating awareness and increasing enrollment and utilization of RSBY among the BPL HHs are not consistent.
• The enrollment agencies were not properly trained, and so they provided very superficial information about the RSBY.
• The insurance companies relied on local governance structures for informing the beneficiaries about the scheme. These local health workers often do not view RSBY information dissemination as a priority due to numerous other responsibilities.
• Districts with active political leaders often reported better awareness and enrollment of RSBY. At the same time, it was also mentioned that the powerful and politically connected HHs will receive benefits but will also be well informed about the social protection mechanisms.
• The local political leaders usually neglected the poor people. Tribal and minority groups tend to live in isolation as a result. Isolated living prevented the exchange of information.
• The name of the scheme was also reported as too long for the common man to remember.
• The enrollment agencies usually distributed chits (a small paper mentioning where and when the enrollment camp will be held) to the HHs whose names featured in the 2002 BPL list. As already mentioned, there are many discrepancies in the list resulting in errors in the identification of HHs (especially in the urban areas), which lead to the exclusion of majority of the HHs.
• Short notice to organize the camp and to enroll the beneficiaries was reported as an important implementation issue resulting in exclusion.
• Hurriedly completed enrollment left very little time for the enrollment agencies to interact with and provide adequate information to the beneficiaries.
• Providing mere booklets of empaneled hospitals or information pamphlets does not mean that information has reached the beneficiaries, many of whom are illiterate.
• Unofficial sources of information (for e.g., neighbors and friends) were also one of the main reasons for poor awareness regarding the RSBY scheme. The HHs, who enrolled just because their community members enrolled, received little information from the enrolling agencies.

## Discussion

Analysis using the SPEC-by-step shows that at each step, there is exclusion of many HHs from the scheme. The coverage decreases with each step. The proportion of awareness, enrolled population, and utilization of benefits is quite low, and it is decreasing with each step. Though the awareness, enrollment, and utilization are low among both the groups, the rural HHs are marginally better than the urban areas.

In similar studies in Maharashtra by few other researchers, the enrollment rate was found to be 39%, which is less when compared with the national average. Lower caste HHs (SC and ST) were found to be poorly enrolled. Remote and tribal villages were not enrolled at all. The poor awareness, program design, and schedules of enrollment were seen as primary reasons for low enrollment. At a district level, it was seen that blocks with tribal population reported lower enrollment (15). The enrollment rates in Maharashtra are quoted to be lower with variation across the districts and within the districts (11).

Our study was also analyzed separately to study the effect of social exclusion on enrollment in RSBY. It was found that the HHs that are socioeconomically disadvantaged are less likely to get enrolled in RSBY. The chances of getting enrolled in RSBY can be restricted by social exclusion (19). It is usually expected that the urban areas will have better awareness, enrollment, and utilization compared to the rural BPL HHs. But in our study, the findings are other way. This may be due to the fact that the scheme was first launched in the rural areas. The urban areas also have better availability of health infrastructure than the rural areas. Still, the obvious differences in enrollment rates and retention of HHs in program highlight pro-rural bias in implementation and more exclusion of urban poor.

A similar case study carried out in Karnataka under Health Inc. found that exclusionary processes operate at all steps of implementation of RSBY scheme. As each step is linked with the others, exclusions in one stage have repercussions on other steps. RSBY itself is not capable of addressing these existing exclusionary processes in society (20).

It is also noteworthy to see that the BPL HHs are still forced to use the private health facilities, and it is more prevalent in the rural BPL HHs. This shows poor availability and accessibility of the public health facilities in the rural and urban poor areas even after 65 years of independence, that too in one of the most forward states like Maharashtra. The use of private health care facilities obviously forces BPL HHs toward more out-of-pocket expenditure, catastrophic payments, and/or neglect of the health (4). It has been reported that top-down health insurance interventions with focus on exit strategies will not work out fully in the Indian context. The government must actively facilitate the potential of CHI schemes to emancipate the target group so that they may transform from mere passive beneficiaries into active participants in their health (21).

In a study in Himachal Pradesh, India, majority of the HHs were aware about the scheme and their eligibility. However, when it comes to “know how” and “know where” of using the scheme, only 49% of the respondents were provided with any written literature by enrolling agencies and only 15% respondents received the list of empaneled hospitals (22, 23). In another report from Uttar Pradesh, India, awareness among the enrolled population was low with 42% respondent aware about the scheme and women being less aware (37%) as compared to men (44%) (22, 23). In a study in Karnataka, India, majority of HHs (71%) reported of being familiar with the name and card of RSBY (24). They also reported that among the enrolled cohort, awareness was increasing about the components of scheme. The essential details for using the scheme were not known to users.

A survey carried out in 2010 in the state of Karnataka right after the implementation of RSBY reports that high proportions of the eligible HHs were aware about the scheme (25). A study in Durg District of Chhattisgarh found that majority of the BPL HHs were aware about the purpose of RSBY (84%) and the amount covered (90%),but they lacked understanding of eligibility criteria (27%), validity of the smart card (25%) and total members covered in the scheme (31%). They further report that very few HHs received the information brochure (with the name of hospitals and information on the scheme). Among the HHs that were able to use the scheme, a small proportion (37%) were aware about the amount being blocked (or deduced). This indicates the asymmetry of information and underscores the need of empowering the users (26).

“Access to accurate information” stands as a cornerstone in utilization of services in any of the targeted interventions in health (27), and health insurance programs initiated by the state are no exception. Poor understanding of hospitals empaneled, services covered, and the facilities therein are alarming and calls upon examination of strategies being adopted by the enrolling agencies in the states with RSBY. It is the time to relook into the role of stakeholders involved in informing people about the health insurance scheme and take appropriate actions (28).

There can be many possible reasons for the limited success of RSBY in Maharashtra in both rural and urban BPL HHs. The most important is the poor planning of RSBY as it was planned at the national level without taking different social and cultural factors in Maharashtra, followed by the poor implementation by the Ministry of Labour in Maharashtra. Here, the Ministry of Health was not involved. Poor awareness among all the expected beneficiaries in the community, especially BPL families as well as stakeholders such as implementers, policy makers, etc., is also a quite significant reason. RSBY provides benefits up to only Rs. 30,000 (~500 US$) per year per HH, which is quite less. The design of the scheme allows only five HH members to be enrolled. This resulted in majority of the HHs enrolling the elderly members and thus excluding the younger children and female members (intra-HH exclusion). The renewal of the cards occurred quite infrequently. Due to annual renewal, many HHs were not able to reenroll next year. It was not the HHs who had a choice to decide if they want to reenroll. The most of the sample HHs were not reenrolled in the scheme by insurance companies in the subsequent years. The enrollment was done through a public event through a campaign approach over the period of few days and that was essentially the problem with RSBY. The chances of exclusion also increased with the annual renewal.

The Ministry of Health in Maharashtra started new health insurance scheme, RGJAY, since last few years with better and improved features and benefits. The RSBY was launched in 2008 and then immediately after few years was being gradually closed in favor of RGJAY. It is possible that RSBY did not get sufficient time to settle down.

There are too many schemes running at the national and state levels in India in health as well as non-health field. Even policy makers/implementers are unaware about the features of these schemes. Usually, these schemes are announced just before the elections for the political gains. Even the names of these schemes are quite similar to each other and also changed often. It is likely that simultaneously operating many such schemes is creating confusion for common man. In addition, there is either no or very poor policy and strategy for creating awareness. For example, the enrollment campaigns are the only main ways used for creating awareness about RSBY in Maharashtra.

### Limitations

There are certain limitations of this paper worth mentioning. RSBY is being implemented in Maharashtra since only last 4–5 years, and the fact that RSBY was gradually closing down in Maharashtra might have affected some results. Another limitation might be the definition of awareness being only whether the HHs have seen the RSBY cards or not. The details of awareness were available only for the subsample of enrolled HHs. The survey was not large enough to provide reliable estimates for individual districts. But the findings do represent the views of the population and can be generalized to the urban and rural areas of the entire Maharashtra state.

## Conclusion

Thus, it is seen that the RSBY had not achieved much in Maharashtra. The awareness and enrollment were quite poor. The utilization of the benefits and services from RSBY was very poor even among the enrolled population. RSBY was launched with good intention, but it lacked good planning and implementation. The poor level of enrollments and renewals highlights the need to reconsider the design of the scheme. RSBY used the old BPL list prepared in 2002. This prevented inclusion of many BPL HHs who were not member of that list. There is a definite need to monitor and evaluate currently existing health care financing schemes at all the levels. Delays and irregularities in ensuring the coverage defeat the objective of providing SHP to the vulnerable groups.

Rashtriya Swasthya Bima Yojana scheme in Maharashtra as well as the other states of India and similar state-sponsored health insurance schemes should ensure sufficient enrollment by proactively educating the vulnerable sections. The scheme should ensure automatic renewal or renewal for maximum duration possible of all the members of the family. It is rational to invest on infrastructure that will provide information support before enrollment, during enrollment, and post-enrollment period to the vulnerable HHs. It is essential to monitor such schemes at the levels of insurers, enrolling agencies, as well as service providers. This can be done at a decentralized level with the involvement of civil society.

Many other states in India are running both RSBY and their state-specific health insurance schemes. But in Maharashtra, RSBY has been suddenly discontinued as the new state-specific scheme RGJAY has been initiated by the health ministry. RGJAY mainly caters to tertiary level healthcare facilities. RSBY was taking care of secondary level healthcare facilities that too within unorganized BPL HHs. So, authorities can think about continuing RSBY in Maharashtra in collaboration with the health ministry, with modified focus and good and improved strategy along with RGJAY.

At the end, it must be mentioned that we cannot only depend upon health insurance schemes to improve the health situation and achieve universal health care in the Maharashtra state and the country. These schemes mainly take care of secondary prevention, i.e., diagnosis and treatment of certain conditions. Indian population, especially socially excluded vulnerable groups, requires primary prevention in terms of health promotion and specific protection. There is definite need to make overall socioeconomic development with more focus on health. For this, we cannot depend upon the private sector. The government has to play proactive role in this by making available primary, secondary, and tertiary healthcare facilities for the poor.

## Conflict of Interest Statement

The author declares that the research was conducted in the absence of any commercial or financial relationships that could be construed as a potential conflict of interest.
